# Cell-based phenotypic screening of mast cell degranulation unveils kinetic perturbations of agents targeting phosphorylation

**DOI:** 10.1038/srep31320

**Published:** 2016-08-09

**Authors:** Shenlu Qin, Xumeng Wang, Huanwen Wu, Peng Xiao, Hongqiang Cheng, Xue Zhang, Yuehai Ke

**Affiliations:** 1 Program in Molecular Cell Biology, Department of Basic Medical Sciences, Zhejiang University School of Medicine, Hangzhou 310058, China

## Abstract

Mast cells play an essential role in initiating allergic diseases. The activation of mast cells are controlled by a complicated signal network of reversible phosphorylation, and finding the key regulators involved in this network has been the focus of the pharmaceutical industry. In this work, we used a method named Time-dependent cell responding profile (TCRP) to track the process of mast cell degranulation under various perturbations caused by agents targeting phosphorylation. To test the feasibility of this high-throughput cell-based phenotypic screening method, a variety of biological techniques were used. We further screened 145 inhibitors and clustered them based on the similarities of their TCRPs. Stat3 phosphorylation has been widely reported as a key step in mast cell degranulation. Interestingly, our TCRP results showed that a Stat3 inhibitor JSI124 did not inhibit degranulation like other Stat3 inhibitors, such as Stattic, clearly inhibited degranulation. Regular endpoint assays demonstrated that the distinctive TCRP of JSI124 potentially correlated with the ability to induce apoptosis. Consequently, different agents possibly have disparate functions, which can be conveniently detected by TCRP. From this perspective, our TCRP screening method is reliable and sensitive when it comes to discovering and selecting novel compounds for new drug developments.

Various immune cells are involved in allergic responses and immediate hyper sensitivity reactions, of which mast cells are at the center^1^,^2^,^3^. Mast cells are mainly distributed in the site throughout the contact surface with the external environment, such as intestine, airways, and skin, where allergic responses mostly occur^4^,^5^,^6^,^7^. After activation, mast cells rapidly and selectively release multiple mediators including cytokines, chemokines, preformed granule-associated mediators and newly synthesized lipid mediators. These mediators exert their functions through diverse mechanisms, for example, killing pathogens directly, recruiting effector cells, or altering the permeability and functions of blood vessels nearby^5^,^6^.

Mast cell activation starts from the binding of multivalent antigen to FcɛRI-bound IgE. Then, the receptors crosslink, eliciting the downstream signal cascades^8^. Hitherto, numerous studies infer that two subunits of FcɛRI, β and γ chains, initiate two interdependent series of cellular signal transduction^9^. The indispensable activation pathway, initiated by the γ chain, starts from the phosphorylation of Syk. Then Src family kinases and PLCγ form macromolecular signaling complex with adaptors such as GRB2, and as a consequence, increase mobilization of calcium^9^,^10^,^11^. The complementary pathway, induced by the β chain, depends on the Fyn-Gab2-PI3K axis and amplifies the signals of the main pathway^9^,^12^,^13^,^14^. It is obvious that reversible phosphorylation plays a pivotal role in those molecular events. Therefore, kinases and phosphatases are attractive targets for developing novel drugs in respect to mast cell degranulation- related diseases. However, regular assays such as β-hexosaminidase release assay, used to detect the perturbations caused by agents, are either single point assays or endpoint assays measuring the cumulative release of mediators. Their limitations regarding real-time and sensitive analysis make them unsuitable for high-throughput screening.

The living cell morphological profiling, based on impedance measurements can dynamically monitor the cellular response to treatments, producing dynamic TCRP patterns. This novel approach can also capture the transitory process of ligand and receptor combination and the activation of downstream signals followed by immediate biochemical and cellular changes. In this work, we used TCRP to address the limitations of conventional methods in analyzing IgE-mediated mast cell degranulation. Because of its ability to dynamically assess and compare the interferences of various compounds, TCRPs from a library containing 145 protein tyrosine kinase/phosphatase (PTK/PTP) inhibitors were monitored. The biological effects on mast cell degranulation induced by these inhibitors were clustered according to their TCRP similarities. We particularly focused on agents targeting the same signal molecule in order to analyze their differences. Syk is a tyrosine kinase located at the upstream of signal transduction, and its inhibitors were found all impeded mast cell activation. Shp2, a tyrosine phosphatase, has been reported to regulate the degranulation through Fyn and Ras^15^,^16^, while only PHPS1 and DCA displayed effective inhibition. Recently, a role for transcription factor Stat3 signaling in mast cell degranulation has been revealed^17^,^18^. However, we found that JSI124, a new and highly-anticipated Stat3 inhibitor^19^, exhibited a totally different TCRP compared with AG490^20^, S3I201^21^ and Stattic^22^. Further studies identified that JSI124 induced the apoptosis of mast cells instead of blocking the degranulation as Stattic, confirming the reliability of our TCRP method.

Altogether, we first established the IgE-mediated TCRP for functional monitoring of mast cell degranulation, providing the possibility for further molecular compounds screening. After screening, two Stat3 inhibitors (JSI124 and Stattic) caught our attention, as the TCRPs of JSI124 and Stattic are distinct although they targeted the same enzyme. Finally, we found that JSI124 induced the apoptosis of cells while Stattic inhibited mast cell degranulation. JSI124 and Stattic were typical examples which verified the sensitivity and reliability of our IgE-mediated TCRP. Consequently, through analyzing the TCRPs, eliminating unsuitable and ineffective agents in the drug development can be more efficient.

## Results

### IgE-mediated TCRP for functional monitoring of mast cell degranulation

**Figure 1:**
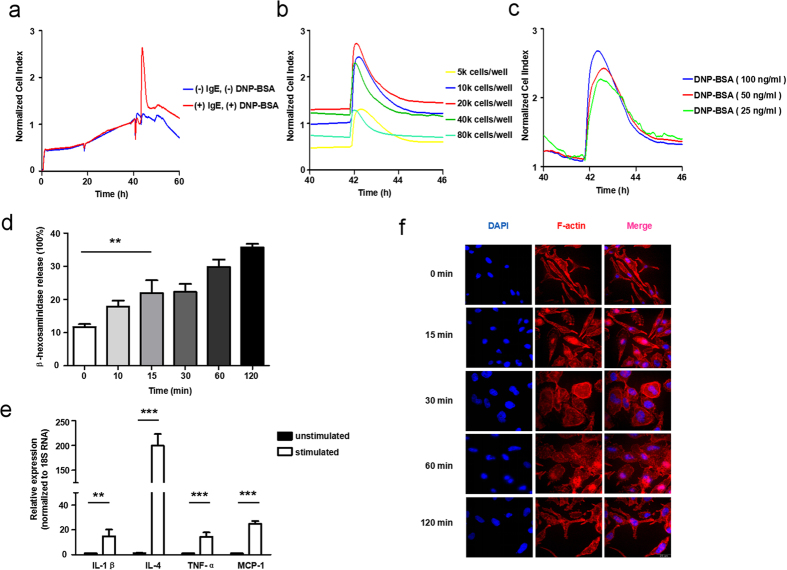
Construction of IgE-mediated mast cell degranulation TCRPs. (**a**) The real time cellular analyzer xCELLigence captured a characteristic peak induced by DNP-BSA. RBL-2H3 cells were seeded overnight and then sensitized with IgE for 24 hours, before the stimulation of DNP-BSA. (**b**)20 k cells per well was the optimal condition for cell culture. Initial inoculums of 5 k, 10 k, 20 k, 40 k, 80 k cells per well were introduced in a 96-well E-plate. Fixed concentration of stimuli was added. (**c**) The reaction to DNP-BSA followed a dose-dependent manner. 20 k cells per well were seeded and treated with different concentrations of DNP-BSA. (**d**–**f**) Mast cell degranulation was coupled with mediators release and morphological dynamics. RBL-2H3 cells were sensitized with 100 ng/mL IgE and activated by 100 ng/mL DNP-BSA. The percentage of released β-hexosaminidase was measured at the indicated time. mRNA levels of cytokines and chemokines were evaluated by qPCR. Morphological changes were detected with LSFM as described in methods. Red: F-actin, blue: nucleus, scale bar = 25 μm. Data are mean ± SD and are representative of three independent experiments. *P < 0.05; **P < 0.01; ***P < *0.001*.

Cell-electrode impedance analysis has been used to reflect changes in cell biological functions in reaction to chemical treatments in previous reports^23^,^24^,^25^,^26^. In mast cells, after stimulation with an antigen, the crosslinking of high-affinity IgE receptor (FcεRI) results in the secretion of vesicles and dramatic remodeling of cell cytoskeleton^4^,^5^,^7^, which can be detected using TCRP. Figure 1a showed a TCRP produced by mast cells in response to DNP-BSA. DNP-BSA stimulation induced an immediate peak of cell index (CI) values after one hour, followed by a decline until reaching a relatively stable level. To investigate this process further, gradual concentrations of cells were seeded into a 96-well E-plate and stimulated by applying the same dose of stimuli. The CI curves generated for different numbers of cells had similar profiles, but showed a shift (Fig. 1b). Since too many and too few cells in culture both resulted in a decrease of peak height, we selected 20 k cells per well as the condition for subsequent experiments. Next, after activating the previously established amount of RBL-2H3 cells with increasing concentrations of DNP-BSA, we observed a dose-dependent effect. The CI values all peaked approximately one hour after DNP-BSA addition and gradually declined to comparable levels (Fig. 1c). The amount of released β-hexosaminidase, which is the preformed granule-associated mediator and the hallmark of degranulation^27^, increased substantially within 15 minutes upon mast cell activation (Fig. 1d). Furthermore, the mRNA levels of several related cytokines and chemokines were also dramatically enhanced (Fig. 1e). Regarding morphological changes of mast cells upon activation, we observed pseudopodium extension and membrane ruffling, which are indicative of cytoskeletal rearrangement. Corresponding to the rise, peak and decline phases of the TCRP, the cell size varied remarkably along with time. The most dramatic morphological changes appeared between 30 minutes and 60 minutes after activation, returning to the initial state after 120 minutes (Fig. 1f). Taken together, cell-electrode impedance-based IgE-mediated TCRP correlates with mast cell degranulation process.

### High-throughput screening and functional analysis of kinetic mast cell reactions to agents targeting phosphorylation

**Figure 2:**
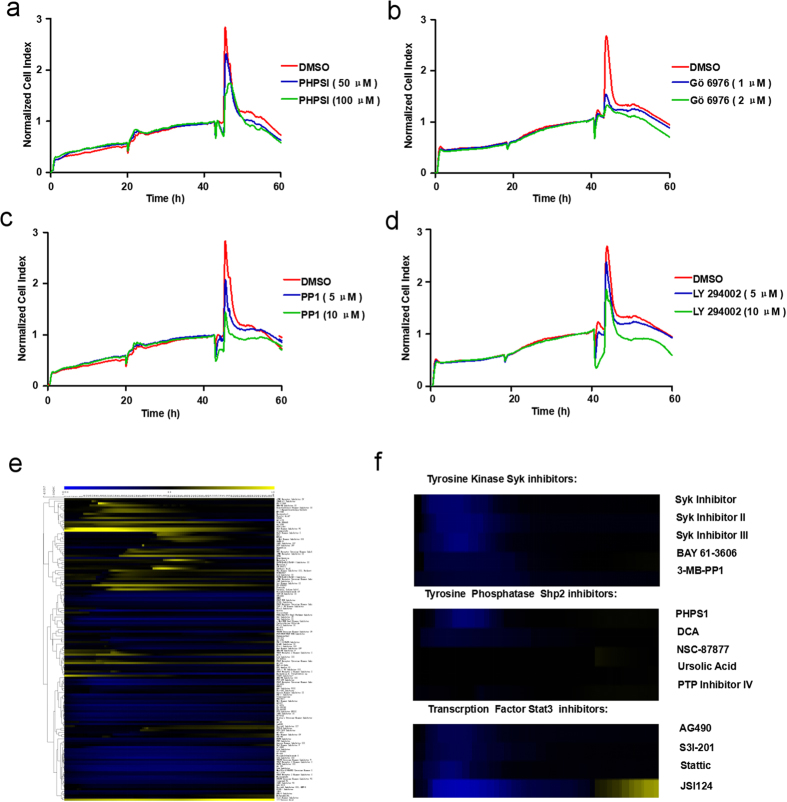
Functional screening of agents targeting phosphorylation based on their TCRPs. (**a**–**d**) Inhibitors of FcεRI receptor activated downstream signal pathways significantly attenuated IgE-mediated TCRPs. IgE sensitized RBL-2H3 cells were pretreated with serial concentrations of inhibitors PHPS1 (**a**), Gö 6976 (**b**), PP1 (**c**) or LY 294002 (**d**) for 2 hours before DNP-BSA stimulation, cell status was monitored for 60 hours. (**e**) Agglomerative hierarchical clustering analysis of kinetic perturbations of agents targeting phosphorylation. Expression level scores were mapped to colors from blue (z = −3.0) to yellow (z = 1.0). Inhibitors that had similar functions were subgrouped together. (**f**) A simultaneous comparison between several inhibitors of the same signal molecule.

Since activated mast cells can produce featured TCRP mirroring their processes of degranulation, we wondered whether inhibiting mast cell activation would affect this pattern. Aggregation of FcεRI receptors results in subsequent stimulation of downstream signaling pathways invovling the phosphorylation and dephosphorylation of various signal proteins^9^. Therefore, we accordingly pretreated IgE-sensitized RBL-2H3 cells with some reported functional compounds: PHPS1(an inhibitor of Shp2^15^,^28^), Gö 6976(a highly selective PKC inhibitor^29^,^30^), PP1(a Src family kinase inhibitor^31^,^32^) and LY 294002(PI3K inhibitor^33^,^34^). We then stimulated mast cells with DNP-BSA and monitored degranulation using cell-electrode impedance measurements. Compared with mast cells treated with DMSO, PHPS1(Fig. 2a), Gö 6976(Fig. 2b), PP1(Fig. 2c) and LY 294002(Fig. 2d) significantly inhibited mast cell activation in a dose-dependent manner, resulting in milder CI increase, as well as lower CI peak values. Therefore, this approach can be used to carry out further high-throughput screening of mast cell degranulation-target inhibitors, with high sensitivity. We next applied our TCRP methodology to measure the kinetic perturbations engendered by an inhibitor library containing 145 PTK/PTP inhibitors, on mast cells. A heatmap, shown in Fig. 2e, was obtained describing mast cell reactions to these inhibitors according to their TCRPs. We chose several inhibitors targeting the same enzyme and compared their functions from the intuitive information provided by the heatmap. All the inhibitors of Syk interfered with the degranulation process, while among the Shp2 inhibitors only PHPS1 and DCA fomented the impedance. Interestingly, compared with other agents against the phosphorylation of Stat3, JSI124 showed a distinctive pattern (Fig. 2f). When considering collectively the information obtained from their TCRPs, we conclude that this technique offers the opportunity to globally identify the functions of multiple chemicals, and accurately distinguish the influence of several compounds targeting the same molecule during mast cell degranulation.

### Two Stat3 inhibitors, Stattic and JSI124, influence degranulation differently as demonstrated by their disparate TCRPs

**Figure 3:**
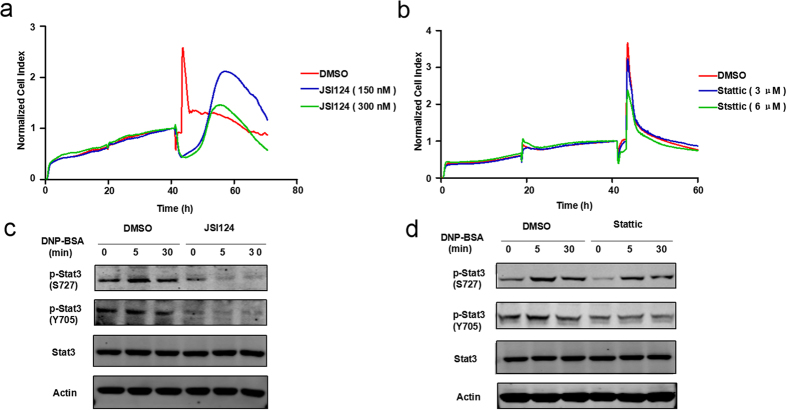
Two Stat3 inhibitors (JSI124 and Stattic) display distinct TCRPs. (**a**,**b**) Unlike for Stattic, the TCRP of JSI124 pretreated RBL-2H3 cells did not yield a typical inhibition curve. (**a**) IgE sensitized RBL-2H3 cells were pretreated with serial concentrations of JSI124 2 hours before DNP-BSA stimulation, cell status was monitored for 70 hours. (**b**) Sensitized cells were pretreated with indicated concentrations of Stattic before DNP-BSA stimulation as (**a**). (**c**,**d**) JSI124 and Stattic both inhibited Stat3 phosphorylation. (**c**) After sensitization, RBL-2H3 cells were treated with DNP-BSA (100 ng/mL) for indicated times. Whole cell extracts were subjected to immunoblotting with anti-p-Stat3, Stat3 and Actin antibodies, cell status was monitored for 60 hours. (**d**) Procedures are the same as in (**c**), except that mast cells were pretreated with 6 μM Stattic for 2 hours.

**Figure 4:**
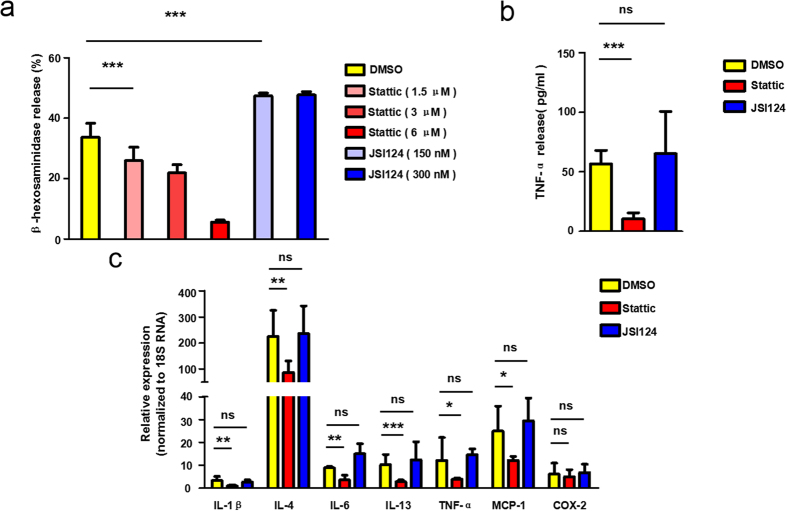
Stattic and JSI124 influence degranulation differently as demonstrated by their TCRPs. (**a**) Stattic inhibited the β-hexosaminidase secretion during mast cell activation, while JSI124 did not. RBL-2H3 cells were sensitized with 100 ng/mL IgE overnight, followed by a 30-minute DNP-BSA challenge. 2 hours before the addition of DNP-BSA, the indicated concentrations of JSI124 or Stattic was added to the medium. β-hexosaminidase releases were then measured as described in the methods. (**b**,**c**) For the cytokines expression and release, Stattic generated an inhibition effect, unlike JSI124. RBL-2H3 cells sensitized overnight were then treated with 300 nM JSI124 or 6 μM Stattic. 2 hours later, cells were activated by exposing them to 100 ng/mL DNP-BSA for 1 hour. TNF-a release and cytokines expression were measured as described in the methods. Data are mean ± SD and are representative of three independent experiments. *P < 0.05; **P < 0.01; ***P < *0.001*.

The phosphorylation of mitochondrial Stat3 is essential for immunologically mediated degranulation of primary mast cells and RBL-2H3 cells through induction of OXPHOS activity^17^. From our screening results, Stat3 inhibitors all displayed inhibition patterns except JSI124 (Fig. 2f). JSI124 is a newly identified Stat3 inhibitor. Its suppression effect on Stat3 phosphorylation levels results in the inhibition of Stat3 DNA binding and Stat3-mediated gene transcription^19^. Inhibition of Stat3 signaling in mast cells leads to impaired FcɛRI-mediated proximal and distal signaling, as well as reduced degranulation^18^. However, the CI curve of JSI124 on mast cell degranulation differed from the anticipated inhibition pattern. Figure 3a showed that JSI124 pretreated RBL-2H3 cells did not respond to the stimulation by DNP-BSA as their CI values decreased dramatically while the CI values of DMSO-pretreated cells reached their peak. When the CI value of DMSO group gradually returned to a steady state, that of the JSI124-pretreated group started to increase vigorously before dropping down fast. We then obtained the TCRP curve of another Stat3 inhibitor Stattic^17^,^22^. Compared with JSI124, Stattic inhibited the degranulation significantly in a dose-dependent manner (Fig. 3b). Moreover, immunoblotting results proved that JSI124 was able to suppress Stat3 phosphorylation without altering the overall Stat3 protein levels (Fig. 3c), and that both JSI124 (Fig. 3c) and Stattic (Fig. 3d) blocked the phosphorylation of Stat3 at Y705 and S727. We subsequently performed regular end-point assays to confirm that their distinctive TCRPs reflected two different functions on mast cell degranulation. On one hand, the results showed that Stattic significantly abolished cytokines expression, TNF-α secretion, as well as β-hexosaminidase release in a dose-dependent manner (Fig. 4a–c). On the other hand, JSI124 neither affected the secretion of TNF-α nor hindered the expression of other cytokines. β-hexosaminidase secretion assays showed that mast cells pretreated with JSI124 released even more β-hexosaminidase than activated DMSO-pretreated cells (Fig. 4a–c). Taken together, the different TCRPs of JSI124 and Stattic reflect their disparate effects on mast cell degranulation.

### The TCRP of JSI124 is reflective of its apoptosis-inducing effect

**Figure 5:**
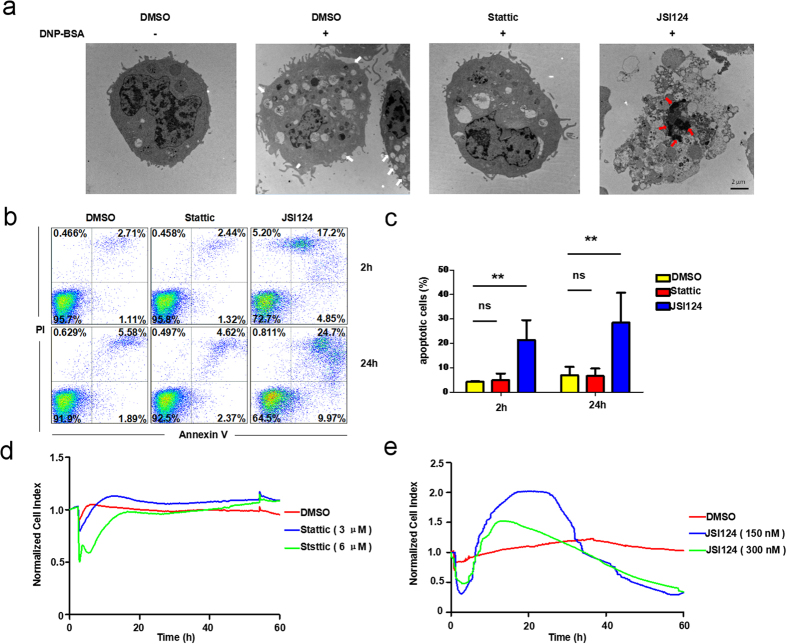
JSI124 induces apoptosis in RBL-2H3 cells. (**a**) Mast cells treated with JSI124 exhibited the characteristics of apoptosis. RBL-2H3 cells were treated with DMSO, Stattic (6 μM) or JSI124 (300 nM) for 2 hours and stimulated with DNP-BSA for 15 minutes before imaging by transmission electron microscope (TEM). Representative images of cells are shown. White arrows indicate membrane fusion; Red arrows indicate chromatin condensation. (**b**,**c**) Annexin V and PI double staining assay illustrated that JSI124 promoted the apoptosis of mast cells. (**b**) RBL-2H3 cells were treated with inhibitors for 2 hours or 24 hours. Cells were stained with FITC-labeled annexin V and PI and analyzed by flow cytometry as described in Materials and Methods. (**c**) The percentage of apoptotic cells was calculated as follows: 100% - the percentage of double negative cells. (**d**,**e**) TCRP of JSI124 was apoptosis-related. RBL-2H3 cells were seeded in the plate for 24 hours, and the medium was subsequently replaced with serum-free fresh DMEM. After the addition of inhibitors, a typical dynamic cell response curve was recorded to detect cell apoptosis.

Since the specific TCRP of JSI124 was different from the inhibiting pattern of Stattic, we decided to find out the biological effects JSI124 caused on mast cells. JSI124 is discovered as an inhibitor with potent antitumor activity due to its ability to inhibit cell proliferation and induce apoptosis^19^. A previous study shows that 150 nM of JSI124 can effectively block Stat3 signaling and initiate apoptosis in CD133-positive NSCLC cells^35^. JSI124 induces suppression of serine 727 phosphorylation of Stat3, leading to cell-cycle arrest through alterations in gene transcription and promoting cell death in a caspase-independent manner in many normal and cancer cells^36^,^37^,^38^. Of note, different levels of p-Stat3 in cells may have an influential role in dissecting two different cell deaths when treating them with JSI124. Cells harboring excessively abundant Stat3 activity may favor apoptotic initiation upon JSI124 treatment, while in normal or many cancer cells harboring relatively lower basal levels of Stat3, ROS generation induced by JSI124 is sufficient to trigger autophagy^39^. Since the immunoblotting results implied that the basal Stat3 level of inactivated RBL-2H3 cells was not low (Fig. 4c,d), we suspected that the TCRP of JSI124 potentially correlated to the function of inducing apoptosis. We therefore examined the intracellular structure of mast cells through transmission electron microscopy. As shown in Fig. 5a, RBL-2H3 cells treated with DNP-BSA reveal the characteristics of activation, manifested by the numerous trafficking vesicles and membrane fusions (white arrows). In contrast, when cells were pretreated with Stattic, the number of vesicles declined dramatically suggesting the blockage of degranulation. Nevertheless, JSI124-treated cells were morphologically distinct, and exhibited apoptotic features such as the appearance of apoptotic bodies and chromatin condensation (red arrows). To provide further evidence, cell death was analyzed through annexin V and propidium iodide (PI) double staining using flow cytometry (Fig. 5b). Treatment with JSI124 results in a significant increase of early phase apoptosis cells (annexin V^+^PI^−^) and late phase apoptosis cell population (annexin V^+^PI^+^) (Fig. 5b,c). These findings clearly indicate that JSI124 induced apoptosis on mast cells. However, at this stage, the confirmation that its distinctive TCRP is directly related to the cell apoptosis process needed to be obtained through further investigation. In a previous study, a typical dynamic cell response curve depicting apoptosis of A549 cells has been established^24^. Following a similar approach, we monitored the cell response curves after the addition of Stattic or JSI124. We compared this apoptotic-related TCPR of A549 cells with patterns produced by Stattic or JSI124 pretreated RBL cells. Expectedly, JSI124, but not Stattic pretreated RBL cells displayed an apoptotic-related TCRP (Fig. 5d,e). Accordingly, we suggest that JSI124, inversely to Stattic, induces mast cell apoptosis instead of inhibiting degranulation and that a typical TCRP, indicative of the apoptosis process, is obtained following the addition of JSI124. These two distinct TCRPs correlate with the different roles of Stattic and JSI124 in mast cells.

## Discussion

Using innovative *in vitro* approaches is essential in drug discovery. It has been estimated that the proportion of cell-based techniques represents over 70% of all assays developed towards early drug screening and discovery. The predictive information provided by cell-based assays is in favor of the hit-to-lead optimization and helps saving the further development costs on candidate compounds^40^,^41^,^42^. In addition, the application of high-throughput screening assays is also of primary importance for a better prediction in drug discovery. Nowadays, a novel label-free cell-based technology called TCRP is receiving more and more attention in cell biology and drug discovery. Compared with the regular endpoint assays, the advantages of label-free detection include simple noninvasive measurement, kinetic measurement, homogeneous assay format, less interference with normal cell function, and reduced time for assay development. Recent applications of the TCRP method using xCELLigence platform include, for instance, monitoring host cellular responses to salmonella infections^25^, detecting epithelial barrier functions^26^ and analyzing compound cytotoxicity^43^. To our knowledge, there have been very limited publications dealing with the preliminary specific screening of the anti-allergic drugs. The objective of this study is to further assess the utility and reliability of impedance-based TCRP on drug discovery in the field of mast cell degranulation associated diseases.

The degranulation of mast cells correlated with morphological changes and mediator release in a short time (Fig. 1d–f), generating a dramatic increase in the electrical impedance recorded by the xCELLigence system. A derived parameter, termed cell index (CI), accordingly formed a signal peak when stimulating with DNP-BSA (Fig. 1a–c). We pretreated a mast cell line (RBL-2H3 cells) with agents targeting phosphorylation, and subsequently monitored their TCRPs to determine their functions. We found that compounds inhibiting mast cell degranulation formed lower TCRP peaks, and reciprocally. Based on our results, this TCRP technique can be used in large-scale screening of novel and functional anti-allergic drugs (Fig. 2).

During the molecular inhibitors screening process, some interesting results caught our attention. The pivotal role of Stat3 phosphorylation in mast cell degranulation recently became a widespread concern. JSI124 and Stattic were both Stat3 inhibitors (Fig. 3c,d), however, their TCRPs on mast cell degranulation were divergent (Fig. 3a,b). Regular endpoint assays identified that Stattic, rather than JSI124, blocked the degranulation process as suggested by their TCRPs (Fig. 4). Further studies proved that JSI124 failed to inhibit mast cell degranulation, against our expectations, but induced the apoptosis of RBL cells, showing a typical TCRP apoptosis pattern (Figs 4 and 5). Moreover, the TCRP of Jak2/Stat3 inhibitor AG490 confirmed its significant inhibition property on mast cell degranulation (Fig. 2f). However, the amount of released β-hexosaminidase after stimulation remained unchanged under AG490 treatment (data not show). A previous study concludes that AG490 pretreatment does not influence mast cell activation by affecting the secretion of the enzyme, but through inhibition of LTC_4_ release^44^. Therefore, this proves that the TCRP method can provide complete conclusions about the whole process.

The following statements contain descriptions of performance considerations for IgE-mediated mast cell degranulation TCRP methodology application. Mast cell degranulation includes multiple biological changes, our TCRP method cannot pinpoint which part the inhibitor acted on. Additionally, the cell index can be influenced by changes in multiple cell status such as cell size, cell number, adherence and even cytotoxicity effects of compounds. Thus, these confounding factors need to be controlled with user experience during the operation.

For the evaluation of the leading compounds in drug discovery, many established analytical methods are widely used. The chemical structures and characteristics of these compounds can be identified with analytical methods such as mass spectrometry and gas chromatography, among others^45^. However, the clinical efficacy or adverse reactions of the agents would remain unexplained without identifying their drug efficacy. Many laboratory studies have shown that an evaluation relying on biological functions and roles is indispensable to assess agents, since it can reflect the clinical efficacy and any unknown risks it may pose to patients^45^,^46^. Therefore, finding an efficient and credible bioanalytical method is sorely needed. In drug discovery, novel leading compounds are not only discovered from high-throughput screening, but are also designed for specific targets. Even if the agent has high specificity towards its target and induces strong inhibition, its ultimate effect is not always the expected, introducing a potential complication in drug design. For the detection of anti-allergic agents, regular endpoint assays typically analyze several single times to assess the overall cellular response. However, our IgE-mediated mast cell degranulation TCRP appears to be a powerful and reliable tool for anti-allergic drug discovery because of its capacity to provide a reasonably high throughput, the possibility for real time monitoring of cell responses to agents, and the potential for preliminary prediction of the compounds’ functions at an early stage of drug development.

## Materials and Methods

### Reagents and antibodies

DMEM and fetal bovine serum (FBS) were purchased from HyClone. IgE (D8406) and the substrate of β-hexosaminidase: 4-methylumbelliferyl-N-acetyl-b-D-glucosaminide (N9376) were from Sigma-Aldrich. DNP-BSA (A23018) was from Life technologies. Inhibitor Select 96-Well Tyrosine Kinase and Phosphatase Inhibitor Library IV was purchased from Merck Millipore. Antibodies of Stat3 and Stat3 phosphorylated at Y705 and S727 were from Cell Signaling Technology.

### Cell culture

Rat basophilic leukemia (RBL-2H3) cells were purchased from American Type-Culture Collection (ATCC). Cells were cultured with DMEM containing 10% FBS (Bioind) and 50 U/ml penicillin/streptomycin (Gibco). Cell culture dishes were placed in the humidified incubator at 37 °C in 5% CO2.

### Time -dependent Cell Response Profile (TCRP) detected by the xCELLigence system

TCRP was detected by the xCELLigence System (Roche Applied Science, Basel, Switzerland) as described previously^23^,^25^,^26^. Briefly, 50 μL of fresh DEME medium (10% FBS) was added in 96-well E-Plates to obtain background readings before the addition of 100 μl of RBL-2H3 cell suspension. After incubated at room temperature for 30 minutes, the E-Plates were placed onto the reader in the incubator and continued to record the cell index (CI). Cells were allowed to attach for 18 hours to get stable baseline-readings before IgE administration. After 24 hours, cells were pretreated with agents for 2 hours prior to DNP-BSA stimulation. Cells were monitored every 2 minutes for a total of 60 hours to obtain TCRPs.

### Immunofluorescence assay

RBL-2H3 cells were attached and spread on the glass coverslips in 12-well tissue cultured plates, treated with 1 ml cell culture medium containing 1 μg/ml IgE overnight. After being washed with PBS 3 times, the cells were stimulated with 100 ng/ml DNP-BSA for the indicated time and then fixed with 4% paraformaldehyde for 30 minutes. PBS containing 0.5% Triton X-100 was used to permeabilize cell membrane for 30 minutes. Permeabilized cells were blocked with 5% BSA for 1 hour. Then the cells were stained with phalloidin (Beyotime) for 1 hour at room temperature, washed three times with PBS, counterstained with DAPI for 5 minutes. Photographs of stained cells were captured by the LSM 510 confocal fluorescence microscope (Zeiss).

### Degranulation assay

RBL-2H3 cells were cultured with DMEM (10% FBS) and sensitized with 100 ng/ml IgE for 12 hours in 12-well tissue cultured plates, then washed and cultured in Tyrode’s buffer. After that cells were stimulated with 100 ng/ml DNP-BSA for the indicated time. To detect the total β-hexosaminidase in the cytoplasm, cells were lysed with 0.5% Triton X-100 in Tyrode’s buffer. Culture supernatants and cell lysates were collected and incubated with the substrate for 1 hour, the reaction was stopped with 0.4 M glycine (pH 10.7). The percentage of degranulation was calculated as follows: absorbance of supernatants at 405 nm × 100/absorbance of cell lysates at 405 nm.

### RT-PCR analysis

Total RNA was extracted from cells using RNA extraction kit (Invitrogen) and was reverse-transcribed using the ReverTra Ace qPCR RT Kit (TOYOBO) to generate cDNA. Real-time PCR was performed with the UtraSYBR Mixture Kit (CWBIO) on LightCycler 480 Real-Time PCR Detection System (Roche). Primer sequences are as follows: IL-1β: 5′-CACAGCAGCATCTCGACAAGA-3′ and 5′-CACGGGCAAGACATAGGTAGCT-3′; IL-4: 5′-ACCTTGCTGTCACCCTGTTC-3′ and 5′-TTGTGAGCGTGGACTCATTC-3′; IL-6: 5′-CACTTCACAAGTCGGAGGCT-3′ and 5′-TCTGACAGTGCATCATCGCT-3′; IL-13: 5′-ATGGTATGGAGCGTGGACCT-3′ and 5′-AGCGGAAAAGTTGCTTGGAG-3′; TNF-α: 5′-GCATGATCCGAGATGTGGAA-3′ and 5′-ACGAGCGGGAATGAGAAGAG-3′; MCP-1: 5′-ATGCAGTTAATGCCCCACTC-3′ and 5′-TTCCTTATTGGGGTCAGCAC-3′; COX-2: 5′-TGACTTTGGCAGGCTGGATT-3′ and 5′-ACTGCACTTCTGGTACCGTG-3′; 18 s RNA: 5′-GTAACCCGTTGAACCCCATT-3′ and 5′-CCATCCAATCGGTAGTAGCG-3′.

### ELISA

RBL-2H3 cells were sensitized with IgE overnight. Cells were pretreated with inhibitors for 2 hours and then stimulated with 100 ng/ml DNP-BSA for 30 minutes. Protein levels of TNF-α in cell supernatants after stimulation were evaluated using paired ELISA kits (eBioscience) following the manufacture’s protocol.

### Immunoblotting

IgE sensitized RBL-2H3 cells were pretreated with DMSO, Stattic or JSI124 for 2 hours and then stimulated with 100 ng/ml DNP-BSA for indicated minutes. The same amount of protein was resolved by 8% SDS-PAGE and transferred onto NC membranes. After being blocked for 1 hour, the membranes were incubated with the primary antibody p-Stat3 (S727), p-Stat3 (Y705) and Stat3 respectively overnight at 4 °C. Secondary antibody was the IRDye800 goat anti-Rabbit IgG. Immunoreactive proteins were visualized using the Odyssey Infrared Imaging System.

### Flow cytometry for detecting cell death

After treatment with inhibitors as described in the figure legends, cells were digested with trypsin and collected. For measurement of cell death, cells were suspended in the FITC binding buffer and stained with FITC-labeled PI (Beyotime, C1062) and annexin V (Beyotime, C1062) for 30 minutes. Fluorescence labeling was analyzed by two-color flow cytometry. Annexin V and PI emissions were detected in the FL1 and FL2 channels of a FACSCalibur flow cytometer, using emission filters of 488 and 532 nm, respectively. At least 50,000 cells were analyzed in each of three independent experiments.

### Statistical analysis

Data are presented as means ± SD. GraphPad Software 5.0 was applied to perform Student’s unpaired t test. P < 0.05 was considered to be statistically significant.

## Additional Information

**How to cite this article**: Qin, S. *et al*. Cell-based phenotypic screening of mast cell degranulation unveils kinetic perturbations of agents targeting phosphorylation. *Sci. Rep*. **6**, 31320; doi: 10.1038/srep31320 (2016).
